# Genomic Analysis of Two Cold-Active *Pseudoalteromonas* Phages Isolated from the Continental Shelf in the Arctic Ocean

**DOI:** 10.3390/v15102061

**Published:** 2023-10-07

**Authors:** Chung Yeon Hwang, Byung Cheol Cho, Jin Kyeong Kang, Jihye Park, Stephen C. Hardies

**Affiliations:** 1 Microbial Oceanography Laboratory, School of Earth and Environmental Sciences and Research Institute of Oceanography, Seoul National University, Seoul 08826, Republic of Korea; chung.hwang@snu.ac.kr (C.Y.H.); bccho@snu.ac.kr (B.C.C.); biojin@snu.ac.kr (J.K.K.); jihye1216@snu.ac.kr (J.P.); 2 Saemangeum Environmental Research Center, Kunsan National University, Kunsan 54150, Republic of Korea; 3 Department of Biochemistry and Structural Biology, UT Health, San Antonio, TX 78229, USA

**Keywords:** cold-active, temperate, myovirus, *Pseudoalteromonas*, sediment, Arctic

## Abstract

Cold-active bacteriophages are bacterial viruses that infect and replicate at low temperatures (≤4 °C). Understanding remains limited of how cold-active phage–host systems sustain high viral abundance despite the persistently low temperatures in pelagic sediments in polar seas. In this study, two *Pseudoalteromonas* phages, ACA1 and ACA2, were isolated from sediment core samples of the continental shelf in the western Arctic Ocean. These phages exhibited successful propagation at a low temperature of 1 °C and displayed typical myovirus morphology with isometric icosahedral heads and contractile tails. The complete genome sequences of phages ACA1 and ACA2 were 36,825 bp and 36,826 bp in size, respectively, sharing almost the same gene content. These are temperate phages encoding lysogeny-related proteins such as anti-repressor, immunity repressor and integrase. The absence of cross-infection between the host strains, which were genomically distinct *Pseudoalteromonas* species, can likely be attributed to heavy divergence in the anti-receptor apparently mediated by an associated diversity-generating retroelement. HHpred searching identified genes for all of the structural components of a P2-like phage (family *Peduoviridae*), although the whole of the *Peduoviridae* family appeared to be divided between two anciently diverged tail modules. In contrast, Blast matching and whole genome tree analysis are dominated by a nonstructural gene module sharing high similarity with *Pseudoalteromonas* phage C5a (founder of genus *Catalunyavirus*). This study expands the knowledge of diversity of P2-like phages known to inhabit *Peudoalteromonas* and demonstrates their presence in the Arctic niche.

## 1. Introduction

Bacteriophages, or simply phages, are viruses that infect and replicate within bacterial cells, exerting considerable influence on microbial ecosystems [1]. While phages are known to be highly diverse and widespread across various environments, one intriguing subset that has garnered significant interest in the field of environmental viral ecology is the cold-active bacteriophages [2]. These remarkable viruses exhibit a unique ability to thrive and function in cold environments, often operationally characterized by temperatures of 4 °C or below [2,3].

Polar oceans are natural candidates for the search for cold-active phages, as evidenced by field studies demonstrating the abundance and activity of viruses in these environments [4,5]. While studies regarding the isolation and characterization of cold-active phages have been conducted in polar marine environments, the majority of these studies focused on cold-active phages that are specific to bacteria isolated from aquatic environments such as seawater or sea ice in polar regions [3,6,7,8]. However, owing to constraints in accessing and sampling polar continental shelf sediments, our understanding of the phage–host systems in this specific niche is still largely limited [2].

*Pseudoalteromonas* bacteria exhibit a wide-ranging habitat distribution within the ocean, encompassing from the uppermost layer of ocean water (i.e., sea surface microlayer) to the depths of the deep sea [9,10]. Furthermore, *Pseudoalteromonas* is prominently present in polar waters and entrenched within the depths of deep-sea sediments [11]. This microorganism serves as a pivotal model for investigating adaptations to frigid environments and particle-associated copiotrophs [11,12]. Currently, 13 *Pseudoalteromonas* phage genome sequences from the phage isolates have been deposited in NCBI Genomes (accessed on 16 August 2023), including ϕPM2, ϕH105/1 and ϕRIO-1, which are affiliated with various families such as *Corticoviridae*, *Siphoviridae* and *Podoviridae [13]*. Some *Pseudoalteromonas* viruses were found to be cold-active phages, as indicated by their physiological characterizations [8,14]. In a recent extensive study on *Pseudoalteromonas*-associated phage genomes (totaling 144), a high diversity of 47 viral clusters was unveiled at the genus level, spanning across seven distinct families [15]. It was notable that *Pseudoalteromonas* myoviruses were categorized into larger and smaller genome groups based on a genome size criterion of approximately 40 kb, supported by the shared protein clusters analysis and the genome-content-based network analysis [15]. However, among the *Pseudoalteromonas* myoviruses with relatively small genomes, only two phage isolates, namely *Pseudoalteromonas* phages C5a and Maelstrom, have been identified so far [15].

Here, two *Pseudoalteromonas* myoviruses in the small genome group, ACA1 and ACA2, were isolated together with their host strains from Arctic sediment. Both the phage sequences and host strain sequences were determined. Unexpectedly, the host strain for ACA1 was found to already contain a nearly identical prophage, which will be called proACA1-A. Searching of the sequences of phage ACA2 and proACA1-A versus phage ACA1 indicated that these are nearly identical. Hence, phage ACA1 was subjected to a full range of analysis and annotation, and phage ACA2 and proACA1-A were annotated with reference to phage ACA1. Analysis of phage ACA1 sequences will show that it is related to the prototypical *Enterobacteria* phage P2. P2 is a temperate myovirus with a relatively small genome of 33.5 kb given comprehensive reviews [16,17,18]. Nilsson and Haggård-Ljungquist [17,18] considered 6 and 11 completely sequenced genomes, respectively, to be P2-like, with phages HP1, HP2 and K139 appearing to form a closer cluster to each other than another cluster including P2 itself. This division will play a role in this analysis in that phage ACA1 is more closely associated with the HP1 cluster than with P2 itself. They emphasized relatively little recombinational reassortment in the family and a potential for correlation of the phage descent with vertical descent of the host taxa. Lavigne et al. [19] differentiated a group of 18 P2-like phages from other myoviruses using coregenes and designated them as the subfamily *Peduovirinae*. The coregenes analysis also showed a split between a group containing HP1 and another group containing P2 itself, although this split was not accorded taxonomic status. As of the latest taxonomic release by the International Committee on Taxonomy of Viruses (ICTV) [20], *Peduovirinae* has been promoted to family status, now named *Peduoviridae*, and there are now 43 genera assigned to *Peduoviridae* with completely sequenced representatives deposited in GenBank. Most *Peduoviridae* genera are represented by only one or two genomes.

## 2. Materials and Methods

### 2.1. Study Area and Sample Collection

During the IBRV Araon cruise in August 2011, we collected two sediment gravity cores from the continental shelf in the North Pacific sector of the Arctic Ocean (Table 1). These sediment cores, named ARA02B/01-GC-02 (with a length of 4.7 m) and ARA02B/02-GC-01 (with a length of 3.7 m), were frozen and transported to the Korea Polar Research Institute on land. After 3 months of storage, subsampling was conducted on the frozen stored samples, with 1 cm thickness spanning from the surface to the bottom of each core at approximately 1 m intervals. A section of these subsamples was preserved at 4 °C for further isolation of bacteria and phages.

### 2.2. Isolation and Identification of Bacterial Strains

A sediment slurry of each subsample was resuspended using a sterile NaCl (3%, *w*/*v*) solution and spread on marine agar (MA; Difco Laboratory, Detroit, USA) plates. All plates were incubated at 20 °C under aerobic conditions. Bacterial colonies were then picked and subsequently purified 4 times through streaking on fresh MA. To identify the bacterial strains, the 16S rRNA gene for a single colony was directly sequenced after PCR amplification using primers 27F and 1492R, as previously described [21]. The 16S rRNA gene sequence of each strain was compared with the validly published names of prokaryotes in the EzBioCloud database [22]. Subsequently, the genomic sequences of the host strains for ACA1 and ACA2 were obtained (Section 2.6) and genome-wide measurements were used to further clarify the host taxonomy (Section 2.7).

### 2.3. Isolation of Phages

Enrichment of phages from the sediment subsamples and preliminary screening using a spot test were performed as described [23] with the following procedure. To enrich sediment phages, marine broth (MB; Difco Laboratory, Detroit, USA) was supplemented to the sediment slurry (1%, *v*/*v*). After incubation at 4 °C for a week, bulk phages were recovered through centrifugation (at 10,000× *g*) and filtration using a 0.2 µm pore sized syringe filter. Phage activity was preliminary assayed using a spot test on the bacterial lawn of each isolated bacterial strain. For the host strains showing lysis, a conventional double layer plaque assay based on MA was performed using the bulk phages. A single plaque was picked and purified through four rounds of the plaque assay. Two phage–host systems were established (Table 1) and subjected to the following analyses.

### 2.4. Physiological and Morphological Characterization

The temperature range for growth of host strains was determined by observing the formation of the colonies after streaking on MA. The strains were incubated at 1, 3 and 5–40 °C (in increments of 5 °C) for 1 week under aerobic conditions. The temperature range for plaque formation was determined using the double layer plaque assay, following the same conditions used in the growth experiment of the host strains. Cross-infection between the host strains was tested using a spot test at 25 °C.

The effect of temperature on phage production was investigated as previously described [13]. The host strain HL-ACA1 was infected with ACA1 phage at a multiplicity of infection (MOI) of 0.1. After incubation at room temperature for 20 min, they were incubated at different temperatures (4, 10, 20, 30 and 40 °C) for 6 h before determining the titer using the double layer plaque assay.

The morphology of the purified phages, prepared as described in Section 2.5, was observed under a transmission electron microscope (TEM; CM200, Philips, Andover, MA, USA) after staining with 1% (*w*/*v*) uranyl acetate.

### 2.5. Concentration and Purification of Phages

Phages were obtained from a 1 L overnight culture. To remove bacteria, the culture was first subjected to centrifugation and then further filtered through a 0.22 μm pore size membrane (Millipore, Bedford, TX, USA). Subsequently, phages were concentrated using Amicon Ultra-15 centrifugal filter units (Millipore, Billerica, TX, USA). The purification process followed a previously described method [13], which included a CsCl step gradient performed through ultracentrifugation, followed by an exchange with SM buffer (100 mM NaCl, 8 mM MgSO_4_, 50 mM Tris at pH 7.5) for subsequent use. The purified phage concentrates were stored at 4 °C for preservation.

### 2.6. Genome Sequencing of Phages and Host Strains

Phage genomic DNA was extracted from the purified phage concentrate by using the QIAamp MinElute Virus Spin kit (Qiagen, Hilden, Germany). A sequencing library was constructed with the Accel-NGS 2S PCR-free DNA library kit (insert size, ~300 bp; Swift Biosciences, Ann Arbor, MI, USA) and sequenced by Macrogen, Inc. (Seoul, Republic of Korea), using the Illumina MiSeq platform with a 2 × 300 bp paired-end (PE) format. Raw reads were processed as previously described [24] and were assembled using de novo assembly with default settings in CLC Genomics Workbench version 8.51 (Qiagen, Aarhus, Denmark).

To obtain the genome sequences of host strains, genomic DNA was extracted using the DNeasy Blood and Tissue kit (Qiagen, Aarhus, Denmark) according to the manufacturer’s instructions. Two sequencing platforms were employed for the independent preparations of genomic DNA. Firstly, Illumina MiSeq sequencing was used, following the same procedure as described above for phage genome sequencing. Secondly, Nanopore sequencing was performed using the MinION Mk1C sequencer (Oxford Nanopore Technologies; ONT, Oxford, UK) with the ligation sequencing kit (SQK-LSK109; ONT, Oxford, UK). The bacterial genome sequences were acquired following the method described [25] using a combination of ONT long reads and Illumina short reads.

### 2.7. Genome Annotation

For the genome annotation of phages, open reading frames (ORFs) were predicted using GeneMark (http://opal.biology.gatech.edu/GeneMark/, accessed on 15 March 2020) [26] followed by manual comparison to start codons annotated for homologs found by PsiBlast, HHpred and Glimmer [27]. Preliminary annotation of ORFs was performed using Prokka version 1.11 [28]. Translated ORFs were analyzed using BlastP and PsiBlast searches as previously described [29]. To identify homologs of related phages (Section 2.8) to ACA1 and ACA2, we conducted HHpred searches on the HHpred website (https://toolkit.tuebingen.mpg.de/tools/hhpred) (accessed on 6 June 2023). These searches included the library Uni-Prot-SwissProt-viral70_3_Nov21, which integrates HMMs formed from representative phages in the UniProt database into the global collection of searchable HMMs at the HHpred website [30,31,32].

Promoter prediction used the Neural Network Promoter Prediction tool at https://www.fruitfly.org/seq_tools/promoter.html (accessed on 3 August 2023) confined to the areas for the P_e_ and P_c_ promoters precedented in other P2-like phages [17,18].

For the genome annotation of host strains, we utilized the NCBI Prokaryotic Genome Annotation Pipeline (PGAP) [33]. The presence of prophage was checked by PHASTER [34] and BlastN searches between each phage and its corresponding host genome sequences.

### 2.8. Taxonomic Analysis

A full proteome ViPTree (version 3.6; https://www.genome.jp/viptree/, accessed on 1 August 2023) was constructed [35]. The whole genome tree was also constructed in the Virus Classification and Tree Building Online Resource (VICTOR) using the Genome-BLAST Distance Phylogeny (GBDP) method [36] (accessed on 28 June 2023).

To determine the phylogenetic position of host strains, we performed phylogenetic analyses based on the 16S rRNA gene sequences and the genome sequences of the type strains of bacterial species using the Type Genome Server (TYGS) [37]. Overall genome relatedness index (OGRI) values such as average nucleotide identity (ANI) [38] and digital DNA–DNA hybridization (dDDH) [37] were obtained for all pairwise comparisons of bacterial genome sequences.

### 2.9. P2-like Database

All nucleotide sequences retrieved from NCBI by Entrez query *Peduoviridae*[orgn] AND “complete genome” (accessed on 23 June 2013). These were filtered to remove bacterial genomes and small fragments of phage genomes and duplicate entries. This produced 187 unique genomes. Examination of the ORGANISM field produced 43 genera names, which corresponded to the list of *Peduoviridae* retrievable from ICTV with only a few exceptions. Upon extracting protein sequences to a fasta file, the virus genus name was saved into the definition lines so as to facilitate labeling Blast search results, trees and alignments with the virus genus.

### 2.10. Modular Divergence

For each ACA1 protein, the divergence to ACA2 and *Pseudoalteromonas* phage C5a, as reported by BlastP and above-mentioned in Section 2.9, and the divergence to HP1 and P2, as reported by the HHpred server, was tabulated as percent identity (Table S1). Upon noticing that the antireceptor and the associated diversity generating retroelement had much greater similarity to members of genus *VHMLvirus* than to P2-like viruses, we added BlastP-based identities for those specific genes to the tabulation. Several modules with radically different similarity profiles were noted, and these relationships were reported over the module as the median of the values tabulated, excluding proteins for which there was no measure. The median was used rather than an average to reduce the impact of individual genes interjected into the modules by recombinational reassortment.

### 2.11. Timetree Analysis

Collections of P2-like portal or sheath protein sequences were collected by PsiBlast within the P2-like database (Section 2.8) using the ACA1 portal as the query or the ACA1 sheath and P2 sheath as queries. Those sequences were added to preexisting near-global portal or near-global sheath alignments underlying the portal timetree in Hardies et al. [39] or the sheath timetree in Gonzáles et al. [40], using the UCSC sequence alignment and modeling system as described [39]. Segments of validly aligned sequence were judged using HHpred as described [39]. For portal, the valid alignment was among ACA1 103-318, P2 97-315, T4 225-409. For sheath, the valid alignment was among ACA1 86-310, P2 101-334, T4 354-597. P2-like sequences were extracted from that alignment for inclusion in a timetree as follows: sequences mentioned in Nilsson and Haggård-Ljungquist [17,18] were included, except that prophages were substituted with an actual phage entry of the same sequence and, in some cases, a frame that was misannotated, probably due to a mobile element insertion, was substituted by another phage from the same genus. Additional P2-like sequences were added in an effort to sample the most divergent genera based on a neighbor joining analysis using PAUP [41] on the P2 database contingent alone. Other sequences from the original published trees were retained to establish context, including importing the time scales from those published trees. Timetrees were calculated with MrBayes [42], as described in Hardies et al. [39].

### 2.12. Mass Spectrometry

Mass spectrometry was conducted at the National Instrumentation Center for Environmental Management, Seoul National University. The RP-nano LC-ESI-MS/MS analysis was performed using a Thermo Scientific Q Exactive Hybrid Quadrupole-Orbitrap instrument (Thermo Scientific, San Jose, CA, USA) equipped with Dionex U 3000 RSLCnano HPLC system. Assignment of mass spectra to phage proteins was as described [39].

### 2.13. Nucleotide Sequence Accession Numbers

The annotated sequences of phage ACA1, ACA2 and proACA1-A (a prophage detected in the genome of strain HL-AS1) have been deposited in GenBank with accession numbers OR438928, OR438929 and OR438030, respectively. The GenBank accession numbers for the complete genome sequence of strain HL-AS1 are CP133569 (chromosome) and CP133570 (plasmid); for HL-AS2, they are CP133571 (chromosome) and CP133572 (plasmid). All of these sequences are also available at https://Stephen-Hardies.github.io/ (accessed on 29 August 2023).

## 3. Results

### 3.1. Isolation and Characterization of the Phage–Host Systems

A total of 36 bacterial strains were obtained from the Arctic sediment samples where the bottom water temperature of the seafloor was quite low (−0.7 to 0.7 °C; Table 1). More than half of these strains (*n* = 20) were affiliated with *Pseudoalteromonas* spp., with similarities of 98.8–100% in their 16S rRNA gene sequences. All strains served as host strains for screening specific phages. Two phage–host systems were isolated from subsurface sediment samples beneath the seafloor at depths of 1.9 and 4.0 m on the continental shelf (Table 1). The analysis of 16S rRNA gene sequences of two host strains, HL-AS1 and HL-AS2, revealed that they belonged to the genus *Pseudoalteromonas.* The isolated phages, named ACA1 and ACA2, exhibited infection specificity to their corresponding host strains, respectively. Both *Pseudoalteromonas* phages formed clear plaques of 1–2 mm on double layer agar plates (Figure S1).

The temperature range for growth of strains HL-AS1 and HL-AS2 was 1–40 °C, with optimum growth occurring at 20–30 °C. Based on the characteristics of growth temperature, these can be considered as psychrotolerant bacteria [43]. Plaque formation of ACA1 and ACA2 was observed across the entire range of temperatures tested (i.e., 1–40 °C), including at 1 and 3 °C, which characterizes them as cold-active phages [2]. Phage production showed a gradual increase with temperatures ranging from 4 °C to 30 °C (Figure S2). At 30 °C, phage production was approximately two orders of magnitude higher compared to that at 4 °C, which was the optimal growth temperature of the host strain. Phage production sharply decreased at 40 °C and was approximately 10-fold lower than that at 4 °C (Figure S2).

**Figure 1:**
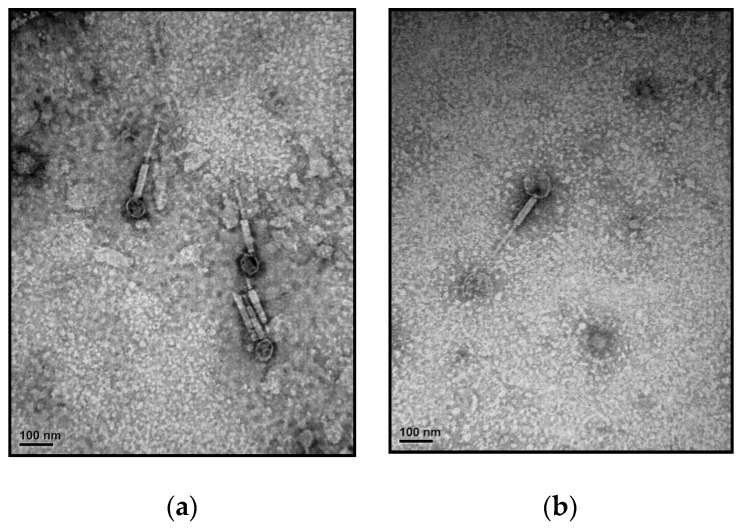
Electron microscopy of virions (**a**) ACA1; (**b**) ACA2. Scale bars represent 100 nm.

TEM observation of ACA1 and ACA2 showed that both were very similar in morphology (Figure 1), displaying an icosahedral head (62 ± 7 nm in diameter) and a contractile tail (120 ± 11 nm in length). Those phages had a typical myovirus morphology.

### 3.2. Taxonomic Classification of Host Strains

**Figure 2:**
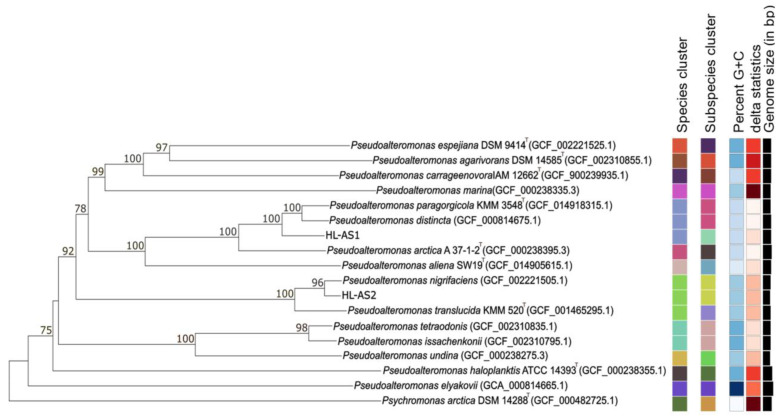
Phylogenomic tree obtained from TYGS [37]. The branch lengths are scaled in terms of GBDP distance formula d5. The numbers above branches are GBDP pseudo-bootstrap support values >60% from 100 replications, with an average branch support of 92.6%.

The 16S rRNA gene sequences of host strains obtained by direct sequencing were not sufficient to specify the *Pseudoalteromonas* species. Based on the 16S rRNA gene sequences, for example, strain HL-AS1 (the host of ACA1 phage) was identical to those type strains of the validly named species *Pseudoalteromonas distincta*, *P. elyakovii*, *P. paragorgicola* and *P. arctica*. Phylogenomic analysis based on the genome sequences revealed that strain HL-AS1 was clearly separated from the nearest clade containing *P. paragorgicola* and *P. distincta*, with high bootstrap support (Figure 2). The dDDH values between strain HL-AS1 and the closest species, *P. paragorgicola* and *P. distincta*, were 72.0–72.4%, which falls within the twilight zone of the bacterial species differentiation cutoff of 70% [44], suggestive of a new candidate *Pseudoalteromonas* species.

Strain HL-AS2 (the host of ACA2 phage) formed a robust clade with *P. nigrifaciens* in the phylogenome tree (Figure 2). A high dDDH value of 89.5% between strain HL-AS2 and the type strain of *P. nigrifaciens* strongly indicated that strain HL-AS belonged to the species *P. nigrifaciens.* Strain HL-AS2 was closely related to strain HL-AS1 with a high similarity of 16S rRNA gene sequences (99.7%). However, a low dDDH value (25.1%) between the host strains confirm that HL-AS1 and HL-AS2 belong to different *Pseudoalteromonas* species, consistent with their respective phages not cross plating.

### 3.3. Sequencing and Relationship to Prophage proACA1-A

**Figure 3:**
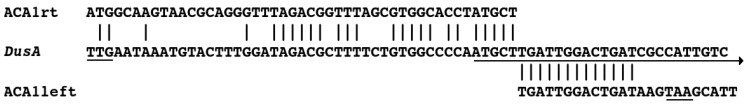
Attachment sequences of proACA1-A. The uninterrupted *dusA* sequence is from the closely related genome of *Pseudoalteromonas* sp. LC2018020214 (CP066804.1). The start codon for *dusA* is variously annotated in *Pseudoalteromonas* genomes either at the underlined ATG or the alternative start codon underlined at left. The frame in the right arm of proACA1-A is open to the alternative start codon. In the circularized genome, the underlined termination codon would truncate this polypeptide.

The complete genome sequence of phage ACA1 was 36,825 bp in size with a G+C content of 43.1%. Phage ACA2 has a genome of 36,826 bp with the same G+C content as phage ACA1. BlastN comparisons revealed that ACA1 and ACA2 were nearly identical and surprisingly also revealed that there was another near identical integrated copy of ACA1, which we have named proACA1-A, in the ACA1 host (strain HL-AS1) genome. Differences among the three sequences are concentrated in the antireceptor. Outside of the antireceptor, there are only six nucleotide differences between ACA1 and proACA1-A and six differences between ACA1 and ACA2. The integration of the prophage is in the host gene for tRNA dihydrouridine(20/20a) synthase (*dusA*; Figure 3). During integration, the right end of the phage appears to reconstruct the 5′ end of the *dusA* gene, implying that transcription of that gene falls under the control of a phage promoter in the lysogen. The latter situation is analogous to that discussed in Karlsson et al. [45] for P2-like phage ϕD145. The GenBank files for ACA1 and ACA2 genomes are reported with the same end points as the prophage.

**Figure 4:**
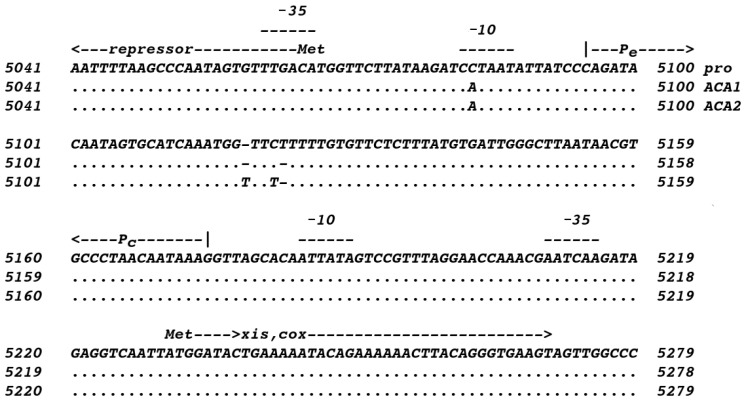
Sequences in the immunity region of proACA1-A, ACA1 and ACA2. Promoters were predicted as in Section 2.6.

The presence of the prophage raises a quandary about how phage ACA1 is able to grow in this host rather than being subjected to immunity. The proACA1-A and ACA1 genomes have only a few differences, but close examination of the read data reveals that at the sites of those polymorphisms, 5–10% of the reads match proACA1-A. Hence, the prophage was not only present during propagation of ACA1; it is partially induced by superinfection by ACA1. Of the very few differences, there are some in the immunity region that may be explanatory. The immunity region of P2 phages features a rightwards promoter (P_e_) that expresses regulators of late transcription and which fires directly into an opposing leftwards promoter (P_c_) that transcribes the immunity repressor [17,18]. That region is packed with binding sites for the repressor and for the product of *cox*, which is the first gene of the rightwards operon and acts to favor the lytic mode. Whereas most P2-like phages start the P_e_ transcript with *cox*, the ACA phages have two paralogs of *cox* there, which we are calling *xis* and *cox* (Figure 4).

One difference shared by ACA1 and ACA2 relative to the prophage is that the TATAA sequence for the P_e_ promoter is closer to the canonical TATAAT, which presumably means that it is stronger and more strongly favors the lytic cycle. The other differences around 5120 are not the same for ACA1 and ACA2. The concentration of changes in that narrow site marks it as hypervariable, and it is likely subject to some selective pressure. We have not inferred repressor or Cox binding sites for these phages as of yet, but we suspect that this site also plays into producing a virulent or partially virulent phenotype. There is no prophage in the host of ACA2. The fact that both ACA1 and ACA2 show variation in this region suggests that this phenomenon is more general than just reflecting the last host encountered, perhaps reflecting an ongoing jockeying of the immunity switch as phages pass through their natural host population in competition with their own siblings.

### 3.4. Genome Annotation

Preliminary analysis by Prokka and PsiBlast searches, as well as a ViPtree whole proteome tree and VICTOR tree analyses (Figures S3–S5) placed phage ACA1 within the *Peduoviridae* family, with its closest relationships being to *Pseudoalteromonas* phage C5a, the founder of the *Catalunyavirus* genus. The founder and most thoroughly characterized phage of *Peduoviridae* is *Enterobacteria* phage P2, and so we sought to annotate the function of each ACA1 protein by establishing which P2 gene product was homologous, if possible. However, because phage ACA1 is in the HP1-like group and there is sometimes significant distance between the HP1-like sequences and the P2 sequences themselves, we found that simple BlastP or even PsiBlast did not always identify the P2 homolog of our ACA1 proteins. To overcome this, HHpred searches were conducted. Matches to HMMs for *Enterobacteria* phage P2 and *Haemophilus* phage HP1 from the SwissProt-viral database were particularly noted. These are listed in the GenBank file, along with matching pdb structure models where available. There are not many structural determinations associated with P2-like phages. But, as noted in Nilsson and Haggård-Ljungquist [18], there are a number of cryoEM determinations of pyocins and other phage-derived bacteria injection systems that are related to the P2-like phages. Additionally, a few of the P2-like tail proteins matched across into other myovirus families by HHpred, and these are also noted in the GenBank file.

### 3.5. General Features of the Genome

**Figure 5:**
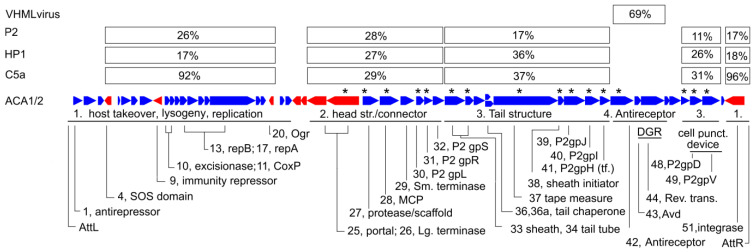
Map of the ACA phage genomes. The genes are assigned to four modules based on variation in the median similarity to *Pseudoalteromonas* phage C5a, *Haemophilus* phage HP1, *Enterobacteria* phage P2, and to members of *VHMLvirus*. Asterisks indicate gene products detected by mass spectroscopy (see Table S2).

Figure 5 shows the features mapped onto the ACA1 genome. By comparison to P2-like phages in general, its most variable regions are the nonstructural genes, and a module including the antireceptor.

The relationships of the ACA phages to other phages of the P2-like family are modular. The similarity of each protein, where detectable, to P2, HP1 and C5a are tabulated in percent identity in Table S1. These tend to show four different patterns, each sustained over a significant number of genes, as indicated by a median percent identity in Figure 5. The close relationship to C5a is confined to the nonstructural proteins. In the head structure and connector module, the average distances of the ACA phages to P2, HP1 and C5a are roughly the same. In the tail structure module, the similarity of HP1 is about twice as great as P2 and C5a is about the same as HP1. Finally, the antireceptor and an adjacent diversity generating retroelement are unlike other P2 family phages and bear similarity to members of *VHMLvirus*.

### 3.6. Head Structure and Connector Module

The head structure module contains several P2-like features. First, it begins with the large terminase subunit gene and portal gene in an inverted orientation. This is the only multigene leftwards operon. As in P2, the portal is followed by two other genes, but they are not homologous between P2 and the ACA phages. The two proteins encoded downstream of portal in the ACA phages are holins. The lysis genes are usually in a module inserted somewhere among the tail genes in P2-like phages, but that module is missing in the ACA phages. The holins are relocated to the inverted segment of the head module, and the endolysin and spanins are as yet unidentified. The remainder of the head structure and head–tail connector genes are in a syntenous arrangement with P2. This includes a characteristic prohead protease/scaffold fusion protein located upstream of the major capsid protein and a small terminase subunit encoded downstream of the major capsid protein. Cleavage sites between protease and the scaffold and of propeptides on the beginning of portal and major capsid protein appear to be conserved, and a semitryptic peptide confirming the portal cleavage was observed (Table S1). The other predicted cleavage sites fall in tryptic peptides that are either too large or too small to be in the range of detection, but the distribution of the detected peptides is consistent with the predicted cleavage. Most notably, in the protease/scaffold fusion predicted to be cleaved into separate protease and scaffold polypeptides, all the detected peptides map to the protease domain, and none map to the scaffold domain. This matches the maturation pattern observed in P2 [46] and documents that there cannot be a large amount of prophage contamination in the preparation subjected to mass spectroscopy.

### 3.7. Portal Timetree

Since the similarities in the head structure region suggest roughly the same time of descent of ACA1, HP1, P2 and C5a from a common ancestor, we asked what time that was. We have initiated an approach of using Hidden Markov Models (HMM) and timetrees to study the descent of phage genes in time [39,47]. The timescale introduced in this approach initially is based on seeking congruency in parts of the tree to a global large terminase tree and assuming that the terminase system was present at the beginning of cellular life on Earth. This assertion was supported in Hardies et al. [39] by demonstrating that it led to correctly assigning the time of passage of the single subunit RNA polymerase into eukaryotes through the mitochondrial endosymbiosis at ~2 Gya.

**Figure 6:**
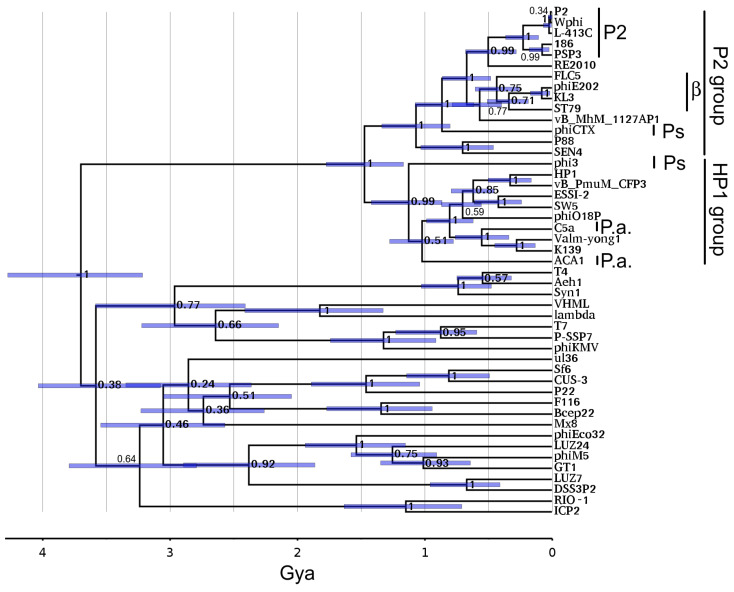
A P2 family on portal timetree. The portal timetree from Hardies et al. [39] was updated as described in Section 2.10. Bars show 95% node height probability interval. Node numbers indicate topological posterior probabilities. Genus and host of P2-like phages used in the timetree analysis are found in Table S3. P.a. marks *Pseudoalteromonas* phages; Ps marks *Pseudomonas* phages; β marks phages from *Betaproteobacteria*; P2 marks phages assigned to *Peduoviruses*. For comparison, *Pseudomonas* is about 1.5 Gya diverged from *Enterobacteria* and betaproteobacteria diverged about 2 Gya from the other hosts shown.

Figure 6 shows the Hardies et al. [39] portal timetree with P2 clusters added. The prior analysis mainly concentrated on portal proteins of podoviruses. The P2 lineage adds as a completely separate lineage at the root and splits into the P2 and HP1 subgroups at ~1.5 Gya. The sequences sampled in this tree were chosen to include the most divergent members from a preliminary neighbor joining tree of the entire sequenced P2-like phage contingent. The subgroups began diversifying around 1 Gya with a strong posterior probability score. Hence, all of the sequences appear to be confidently assigned to one subgroup or the other, although a closer outgroup would be helpful to confirm that. The associations of each P2-like sequence with phage genus and host are given in Table S3. This division into P2 and HP1 subgroups is the same as will be seen with the tail module (Section 3.11). This extent of divergence is typical of what we generally see for the ICTV-defined phage families after their abandonment of the families based on morphological tail types [48]. For example, T7 through phiKMV on Figure 6 defines the envelope of the *Autographiviridae*, and P22 through Sf6 defines the envelope of the *Lederbergviruses*. Nilsson and Haggård-Ljungquist [18] had arbitrarily placed phiCTX at the root of the P2-like phages based on it infecting the most divergent of the host taxa observed at that time. The hope for simplicity of phage and host relationships has somewhat evaporated. Figure 6 indicates that the split into the two groups is the root of the family. *Pseudomonas* is no longer the most divergent host of P2-like phages. The collection marked β on Figure 6 is found in Betaproteobacteria, which is a host ~2 Gya diverged from the others, which are all from Gammaproteobacteria. The connection between the gamma and betaproteobacterial P2-like phages clearly requires a horizontal transfer. A timetree of the large terminase subunit was also constructed and found to closely mirror the properties of the portal timetree (Figure S6).

### 3.8. Time of Residence of Phage Lineages in Pseudoalteromonas

By examining Blast matches to prospective prophages in the sequenced bacterial genomes, it is possible to observe the recent amplification of each of the ACA and C5a phage lineages in *Pseudoalteromonas*. For both ACA phage portal and large terminase, about a dozen BlastP matches appear in *Pseudoalteromonas* genomes down to about 10% divergence; then, there is a gap to the next related lineages found in other host taxa. The C5a portal and large terminase show a similar profile, only with fewer prophages in *Pseudoalteromonas*. Then, a gap to a different host taxon for the next nearest relatives exists. It is known from a clock developed for these genes for a different phage family [39] that the envelope of time encompassed by 10% divergence is on the order of tens of millions of years—probably younger than the lifetime of a host species and certainly younger than the lifetime of a host genus. Whether either of these lineages arrived in *Pseudoalteromonas* at that time by horizontal transfer or had amplified in *Pseudoalteromonas* before that time is unclear because prophages turn over with time. There is only a record of where the common ancestor may have been at any given time if the progenies of two lineages splitting out at that time have been recovered. It is clear that for a few tens of millions of years, the ACA lineage and the C5a lineage were amplifying together in *Pseudoalteromonas*. It is also clear that ACA1 and ACA2, infecting two different *Pseudoalteromonas* species, moved between those species very recently with little sequence change other than in the antireceptor (see Section 3.13).

### 3.9. Nonstructural Module

The nonstructural module shares the general organization and gene content of the other P2-like phages. The orientation is mostly rightwards (in the direction of most of the structural genes), including the replicative repA and B proteins; the characteristic *cox* transcription factor promotes the lytic cycle in P2 and also acts as an excisionase by binding in the attachment site and directing the integrase to operate in the direction of excision. The ACA phages differ in having two paralogs of *cox* at the beginning of the rightwards operon. The first has greater similarity to other excisionases genes, while the second has greater similarity to P2 *cox* itself. Therefore, we presume that these two have divided those two functions. There are a few solo leftwards directed genes in the module. Three of these, like P2 itself, include the immunity repressor, the *ogr* gene product, an activator of late transcription, and the integrase. Many P2-like phages and P2 itself are not SOS inducible, and their repressor lacks the lexA-like autoproteolytic domain that makes the lambda repressor SOS inducible. The immunity repressor of the ACA phages also lacks the autoproteolytic domain, but there is a fourth solo leftwards frame that encodes the domain as a separate polypeptide (gp4). It possibly associates noncovalently with the immunity repressor and renders it SOS inducible. One of the closest HHpred matches to ACA1 gp4, UmuD, functions in an analogous fashion to confer SOS regulation on a cellular function [49]. However, there is another protein encoded that has a connection to SOS induction. Gp1 is homologous to the antirepressor of P2-like phage 186. In that phage, the antirepressor is repressed by cellular LexA itself. When SOS induction inactivates LexA, the antirepressor is expressed, and it functions to inactivate the phage 186 immunity repressor [50]. Since there seem to be two possible immunity regulators, we assume that their functions are differentiated in some way.

### 3.10. The C5a Connection

From Table S1, it is clear that the association with C5a is limited to the nonstructural module and is very close, with a median > 90% identity. The divergence of the ACA nonstructural genes to the other P2-like phages is consistent with independent descent from a more distant time, possibly the same time as the differentiation of the head modules. Assuming the divergence rates of nonstructural genes are similar to the structural genes, the interchange that made the ACA phages and C5a similar in the nonstructural module will have occurred in that same tens of millions of years described in Section 3.8 as the time of observable coresidence of the ACA and C5a lineages in *Pseudoalteromonas*. It may be reasonable to propose that one of these lineages preexisted in *Pseudoalteromonas* and the other arrived by horizontal transfer, aided by obtaining the nonstructural module in a block recombination from the resident lineage. Analyzing this further, it is confounded by a high and variable rate of recombinational reassortment in this module. The 22 proteins of the nonstructural module of phage ACA1 feature 10 with >90% identity, 6 ranging down to 43% identity, and 6 that are apparently unrelated to C5a. Some of the genes, e.g., *ogr* and the gene for integrase, that are highly similar between the ACA phages and C5a also have highly similar homologs scattered around *Pseudoalteromonas* chromosomes in presumptive prophages. Others, e.g., the gp4 gene and *cox*, although highly similar between ACA phages and C5a, are not found to have any close relatives in any prophages in currently sequenced *Pseudoalteromonas* genomes (other than the proACA1-A instance). If one thinks of the collection of prophage genes as a collection of alleles available for acquisition, then to share multiple low frequency alleles as seen in the case of the gp4 gene and *cox* requires that they transferred in one recombination event.

### 3.11. Tail Structure Module

Among the P2 structure proteins that were found by HHpred but not PsiBlast were the contractile sheath, tail tube, tail sheath initiator, baseplate protein P2 gpU, and the cell-puncturing device. For these proteins, the median identity as aligned by HHpred was 33% for ACA1 to HP1, but only 13% for ACA1 to P2. In Table S1, it is seen that all of the tail proteins other than the antireceptor have a similar discrepancy in the relationship to HP1 and P2, whether or not PsiBlast was able to reach across the higher divergence to P2. The division is so marked that even with Blast and PsiBlast, it is possible to find a threshold for each gene that divides all of the P2 genera into a group matching better to ACA1 and a disjoint group matching better to P2. This constitutes an expansion of the HP1 and P2 subgroups previously discussed in the literature; it also corresponds to the split seen in the portal tree (Figure 6), and the whole proteome trees (Figures S3 and S4). Table 2 extends the definition of the HP1 and P2 subgroups to all of the *Peduoviridae* genera currently cataloged in GenBank.

### 3.12. Sheath Timetree

**Figure 7:**
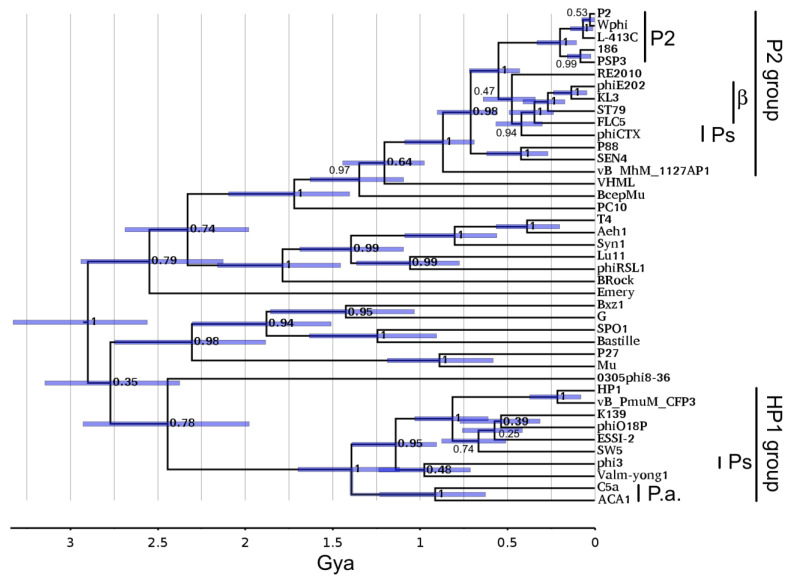
P2 family on sheath timetree. The sheath timetree from Gonzáles et al. [40] was updated as described in Section 2.10.

Since the division of the HP1 and P2 subgroups appeared to be much deeper for tail proteins than for head proteins, we investigated how deep this split was using a contractile sheath timetree. A near-global contractile sheath timetree has been published [40] using the same technique as the portal timetree in Figure 6. Figure 7 shows that sheath timetree updated by adding P2 family sheath sequences from the same P2-like genomes as in Figure 6.

On this tree, the HP1 and P2 subgroup sheath proteins are so different that they merge with the radiation of myoviral tail modules estimated to occur in the 2.5 to 3 Gya range. Within the uncertainty attendant to that time range, it is not determinable if there was a global P2-like ancestor distinct from the other myoviruses. Like the portal tree, within error, the HP1 and P2 subgroups began to diversify at ~1 Gya. For this protein, the ACA phages and C5a do appear to be linked; but this appears to be in the 1 Gya time range around the time of the ordinal host split, not the more recent time inferred for the exchange of the nonstructural gene module. Again, the time of the links to the betaproteobacterial P2-like phages, and probably the *Pseudomonas* P2-like phages, is too recent to represent anything other than horizontal transfer.

The sheath tree shows some non-P2-like viruses related to the P2 subgroup clade. Not far above the diversification of the P2 subcluster lies VHML. VHML is a founder of the *VHMLvirus* genus for which the extensive similarity to the P2 tail module has been noted, although the rest of the genome bears more similarity to lambda-like and N15-like phages [51]. Above VHML lies BcepMu. BcepMu is a phage of the betaproteobacterial host *Burkholderia* and the founder of the *Bcepmuvirus* genus. BcepMu has also been noted to have P2-like tail proteins, although the rest of the genome is described as Mu-like [52]. Above that is *Campylobacter* phage PC10 (GenBank accession of MZ047271). This is an exemplar of an unclassified phage family mostly known through prophage sequences. This sequence is mostly uncharacterized, but Table S4 reveals multiple tail proteins of about the same similarity to P2 tail proteins as the sheath is, but a dissimilar set of head proteins. So, the tail module found in the P2 subgroup has exchanged relatively liberally with other myoviral families in early times down to around 1.5 Gya.

A timetree of the gpJ-like baseplate protein was also constructed and found to closely mirror the results with the sheath timetree (Figure S7).

### 3.13. Antirepressor and Diversity Generating Retroelement

**Figure 8:**
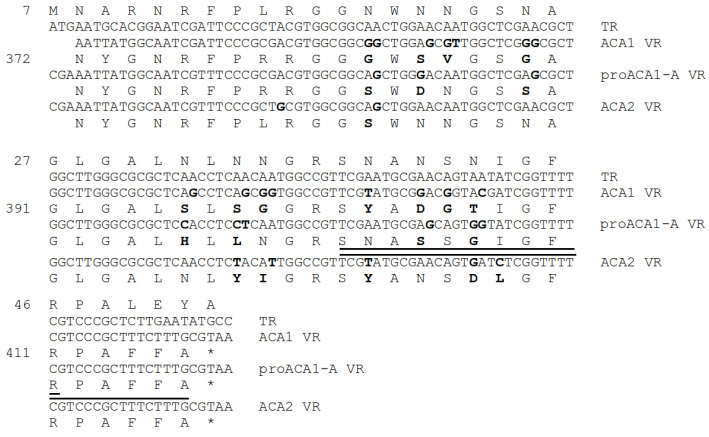
Hypervariability of the antireceptor variable regions. TR is the template repeat which is identical in all three genomes. Numbers on the left are the residue number in the corresponding protein. Variations following the A→N paradigm are in bold. There are some other variations which may result from more ordinary divergence mechanisms. Peptides detected by mass spectroscopy are underlined. Note that the prophage variety is the sequence that was detected, not the ACA1 variety. * indicates a terminator codon.

A feature of the ACA phages not otherwise observed in the P2-like phages is that the apparent antireceptor is modulated by an adjacent diversity generating retroelement (DGR). These are systems that use a reverse-transcriptase-driven hypermutagenesis process to diversify a targeted segment of a protein-encoding gene, called the variable region (VR) [53]. The prototype was the antireceptor of *Bordella* phage BPP-1. The system in the ACA phages is distantly homologous to both the BPP-1 antireceptor and the associated DGR genes and more closely related to a homologous system in the genus *VHMLvirus*. The DGR consists of three elements downstream of the target antireceptor (ACA1 gp42): (1) a specialized protein called the accessory variability determinant (avd; ACA1 gp43), (2) a repeat of the DNA sequence to be mutagenized, called the target repeat (TR), and (3) a reverse transcriptase of the mobile intron II family (ACA1 gp44). In the ACA phages, the TR is translated as an N-terminal extension of the reverse transcriptase, although this is not commonly the case for other DGRs. The mutagenesis process has the specificity of changing some number of As found in the TR into any of the other three nucleotides and copying the mutagenized sequence into the VR of the antireceptor. Most of the nucleotide differences among the ACA phages fall in their respective VRs. The rest of the antireceptor, DGR, and TR sequences are identical among the ACA phages, except that the prophage has a frameshift within the reverse transcriptase and is presumably no longer functional. The differences among the ACA phages in the VR regions are shown in Figure 8.

There is a third protein: ACA1 gp45, which is of unknown function included in the DGR module because it also has higher similarity to genus VHML virus. In P2 and many P2-like phages, the tail fiber (P2 gpH) has an N-terminal domain that binds to the baseplate and a C-terminal antireceptor domain. In the ACA phages, the N-terminal portion is encoded in a separate polypeptide which conforms to the pattern of similarity of the tail module, and the antireceptor domain is encoded as a separate polypeptide having the aforementioned similarity to phages in the genus *VHMLvirus*.

## 4. Discussion

### 4.1. Classification

Although there are selected genes of ACA phages closely related to *Pseudoalteromonas* phage C5a (*Catalyunyavirus*), we find that those represent a very recent transfer of a module with some low frequency phage genes and are not the property of any substantial cluster distributed in *Pseudoalteromonas*. For most of the phage genes, the divergence is greater than or equal to that which separates many assigned genera of *Peduoviridae*. Hence, we think that for consistency with the taxonomy of the other genera of *Peduoviridae*, the ACA phages should be afforded their own genus. Timetrees describing one of the more distantly related C5a-like proteins (repA) and one of the more tightly related C5a-like proteins (integrase) are found in Figures S8 and S9.

### 4.2. History of the P2-Like Phages

The portal and sheath timetrees can be considered a first look at the evolutionary history of the P2-like phages with more data and more advanced software than have been applied before. The much greater divergence of the tail structure genes between the P2 and HP1 subgroups complicates the conception of what the definition of this family is. The nonstructure, head and connector modules appear to adhere to the usual conception of a family as the progeny of some common ancestor. That common ancestor existed around 1.5 Gya. The tail module does not conform to that expectation. Essentially, as the P2-like and HP1-like clades separated, one of them acquired a much older paralog of the same tail module. The HP1 and P2 subgroups can be considered as subfamilies of *Peduoviridae* with the caveat that the subfamily division may be more ancient than the creation of the family itself.

HHpred characterization usually, but not always, matches the HP1 and the P2 subgroup tail proteins to each other before matching them to tail proteins of the other heavily characterized myoviruses, such as T4 or Mu. So, one can conceive of an ancestor around 2.4 Gya that gave rise to both P2-like tail forms but was already distinct from some of the more diverse myoviruses. Thus far, there is not much evidence that those two tail modules recombined much with each other. However, as noted in Section 3.12, one of those forms also made it into phage clusters not having P2-like head or nonstructural genomic properties. That is only known because the construction of the near-global sheath timetree [40] included some sequences that seemed, based on lower power methods, that they might fall in the upper reaches of the tree, and a few of them then fell on the P2 clade. Nothing is currently known about whether the tail module descending into the HP1 subgroup is also recombined into non-P2-like viruses, or whether P2-like head structure or nonstruture modules appearing at 1.5 Gya had predecessors with any P2-like properties at all.

### 4.3. The DGR

The reason that the ACA phages have the DGR is not obvious. The DGR system was originally characterized in a *Bordetella* phage. *Bordetella* undergoes a kind of antigenic variation on its surface that was cited as a motivation for a high intensity variation mechanism affecting the antireceptor [53]. Since then, DGRs have been found more commonly associated with cellular targets rather than phage antireceptors. Phages, after all, have access to recombination to borrow antireceptors from other phages prospering in a given host. And selection to maintain the DGR would require some benefit to using it repeatedly. There are no other DGRs in any of the other P2-like phages characterized as of now. One peculiarity we noticed is that the spectrum counts detected for the antireceptor were quite high and of the prophage variety. There was some cellular contamination detected in the mass spectroscopy. The spectrum counts normalized by molecular weight were greater than that of the major capsid protein and greater than that of ribosomal proteins. That suggests that there may be something more to the story of the antireceptor than we currently understand. Noting that ACA2 represents a very recent move across species lines, and that it is entirely possible that the ACA phages are only a recent acquisition by *Pseuodalteromonas*, it is tempting to speculate that the DGR is powering a rapid horizontal sweep through a new taxonomic niche for these phages.

## 5. Conclusions

This study presented a comprehensive characterization of two novel phages, ACA1 and ACA2, isolated from Arctic sediment samples. The unique characteristics of these cold-active phages, such as their ability to infect psychrotolerant *Pseudoalteromonas* bacteria, their broad temperature range for growth, and cold-active nature, contribute to our knowledge of phage biology and their potential applications. The genomic analysis of ACA1 and ACA2 provided insights into their relationships with other P2-like phages, revealing a modular structure where different parts of the genome display varying degrees of similarity to different phages. The presence of a DGR within the antireceptor gene of ACA1 and ACA2 added an additional layer of complexity to the study. The DGR mechanism, usually associated with bacterial antigenic variation, challenges our understanding of phage evolution and adaptation to diverse host environments. Furthermore, the portal and sheath timetrees introduced a temporal dimension to the evolution of P2-like phages. The distinct divergence patterns observed in the tail and structural genes indicated complex evolutionary histories and raised questions about the origins of these phage lineages and their interaction with other myoviral families.

Overall, the study of ACA1 and ACA2 phages, along with their host strains, has provided valuable insights into the interplay between phages and bacteria in extreme environments. The genomic analysis, taxonomic classification, and evolutionary context of these phages contribute to our understanding of phage diversity, adaptation, and evolution. The presence of the DGR system in ACA1 and ACA2 adds an element of novelty to the study, inviting further investigation into its role in phage–host interactions. This research underscores the dynamic nature of phage–bacteria relationships and highlights the need for continued exploration of these intricate biological systems.

## Figures and Tables

**Table 1:** Isolation source information of two phage–host systems established in this study.

Phage	Host	Date	Sediment Core ID (Location)	Sediment Depth below Seafloor (m)	Bottom Water		
					Depth (m)	Temperature (℃)	Salinity
ACA1	HL-AS1	3 August 2011	ARA02B/02-GC-01 (74.30° N, 167.65° W)	1.9	322	0.7	34.8
ACA2	HL-AS2	2 August 2011	ARA02B/01-GC-02 (73.64° N, 166.51° W)	4.0	112	−1.7	32.9

**Table 2:** Division of *Peduoviridae* into two groups based on similarity among the tail proteins.

HP1 Group	P2 Subgroup	
*Bielevirus* *Catalunyavirus* *Hpunavirus* *Irrigatiovirus* *Irtavirus* *Longwoodvirus* *Phitrevirus* *Playavirus* *Seongnamvirus* *Valbvirus* *Vulnificusvirus* *Yongunavirus* ACA phages (this study)	*Aptresvirus* *Aresaunavirus* *Arsyunavirus* *Baylorvirus* *Bracchivirus* *Citexvirus* *Dagavirus* *Duodecimduovirus* *Eganvirus* *Elveevirus* *Entnonagintavirus* *Felsduovirus* *Gegavirus* *Gegevirus* *Gemsvirus*	*Kapieceevirus* *Kayeltresvirus* *Kisquattuordecimvirus* *Kisquinquevirus* *Nampongvirus* *Novemvirus* *Peduovirus* *Reginaelenavirus* *Reipivirus* *Senquatrovirus* *Simpcentumvirus* *Stockinghallvirus* *Tigrvirus* *Vimunumvirus* *Wadgaonvirus* *Xuanwuvirus*

## Data Availability

Sequences reported here are deposited in GenBank and can be obtained pre-release at https://Stephen-Hardies.github.io (accessed on 5 October 2023).

## Supplementary Materials

The following supporting information can be downloaded at: https://www.mdpi.com/article/10.3390/v15102061/s1, Figure S1: Plaque images of ACA1 (a) and ACA2 (b) on marine agar plates; Figure S2: Phage production at different temperatures of phage ACA1; Figure S3: Segments from full proteome ViPTree with full reference sequence set; Figure S4: Whole proteomic ViPTree confined to just the exemplars used for single gene tree analysis; Figure S5: A whole genome-based VICTOR tree; Figure S6: Timetree of P2-like large terminase proteins; Figure S7: Timetree of P2-like gpJ baseplate protein; Figure S8: Timetree of P2-like repA proteins; Figure S9: Timetree of integrases related to the ACA phages and P2-like phages; Table S1: Relationship of phage ACA1 proteins in % identity to phage ACA2, P2-like phages C5a, HP1 and P2; and to members of genus *VHMLvirus*; Table S2: Mass spectroscopy of ACA1 virions; Table S3: Phage genus and host of P2-like genomes used in the timetree analysis; Table S4: Similarity of selected *Campylobacter* phage PC10 structural proteins to P2 subgroup structural proteins.
