# Supplementary material for: Genomic Analysis of Two Cold-Active Pseudoalteromonas Phages Isolated from the Continental Shelf in the Arctic Ocean
Source: Viruses. 2023 Oct 7;15(10):2061. doi: 10.3390/v15102061 (PMC10612066; doi:10.3390/v15102061)
Supplement: Supplementary file 1 [file viruses-15-02061-s001.zip › viruses-2615558-supplementary.pdf]

Supplementary Materials

Table S1. Relationship of phage ACA1 proteins in % identity to phage ACA2, P2-like phages C5a, HP1 and P2; and to members of genus *VHMLvirus*.

ACA1 gp	Function	P2 homol.	%ID ACA2	C5a homol.	%ID C5a	HP1 HMM[a]	%ID HP1	P2 HMM[a]	%ID P2	*VHML virus*	%ID *VHML virus*
1	Antireceptor		100								
2			100								
3			100								
4	SOS protease		100	gp4	95						
5			100	gp5	97						
6			100	gp6	99						
7			100	gp7	95						
8			100	gp10	59						
9	immunity repressor	gpC	100	gp11	66	P51704	14	2XCJ_B	36		
10	excisionase		100	gp12	51						
11	transcription factor	Cox	100	gp13	98	P51705	13	4LHF_A	18		
12			100	gp14	96						
13	helicase loader	gpB	100	gp15	56	P51707	18	P07696	20		
14	DNA methylase		100	gp17	60	P51715	17				
15			100								
16			100	gp18	92						
17	repl. initiator	gpA	100	gp19	43	P51711	29	Q06419	32		
18			100	gp20	91						
19			100								
20	transcription factor	Ogr	100	gp22	96			P08762	23		
21	transcription factor (focB)		100								
22			100								
23	holin		100								
24	holin		100								

25	portal	gpQ	100	gp24	40	P51717	39	P25480	36		
26	Lg. terminase	gpP	100	gp25	42	P51718	38	P25479	42		
27	protease/scaffold	gpO	100	gp26	27	P51719	31	P25478	29		
28	MCP	gpN	100	gp27	31	P51720	28	7JW1_B	35		
29	Sm. terminase	gpM	100	gp29	22	P51721	25	P25476	18		
30	head completion	gpL	100	gp30	26	P51722	21	P25475	17		
31	tail completion	gpR	100	gp31	26	P51723	23	P36933	17		
32	tail completion	gpS	100	gp32	37	P51724	25	P36934	26		
33	contractile sheath	gpFI	100	gp33	37	P51725	40	P22501	11		
34	tail tube	gpFII	100	gp34	43	P51726	41	P22502	14		
35			100								
36	tail chaperone	gpE	100	gp39	27	P51730	29				
36a	tail chaperone with programmed frameshift	gpE+E’	100								
37	tape measure	gpT	100	gp40	44	P51731	42	O64314	19		
38	sheath initiator	gpW	100	gp41	38	P51732	37	P51768	17		
39	baseplate	gpJ	99	gp42	36	P51733	32	P51767	17		
40	baseplate	gpI	99	gp43	31	P51734	35	P26701	15		
41	tail fiber	gpH	100	gp44	28	P51735	30	P26700	18		
42	antireceptor		97							VHML gp35	61
43	Accessory variability determinant		100							VP58.5 gp19	81
44	reverse transcriptase		100							VP58.5 gp20	75
45			98							VHML gp37	62
46			100			P51737	16				
47		gpU[b]	100	gp45	31	P51738	31	PF06995.14	15		
48	cell puncturing device mounting domain	gpD	100	gp45[c]	34	P51739N[d]	28	P10312	10		
49	tail spike	gpV	100	gp46	26	P51739C	24	3QR8_A	11		

50			100								
51	integrase	integrase	100	gp2	96	P21442	18	P36932	17		

[a]%identities for HP1 or P2 are by HHpred matching to UniProt-SwissProt-viral70_3_Nov21, or Pfam if prefixed with PF, or pdb if the accesion contains an underscore. Otherwise %IDs are as reported by BlastP.

[b]Homology of this protein to P2 gpU is uncertain.

[c]The homolog of P2 gpD was left out of the C5a annotation (33586..34275).

[d]In phage HP1, the homologs of P2 gpD and gpV are fused to a single polypeptide.

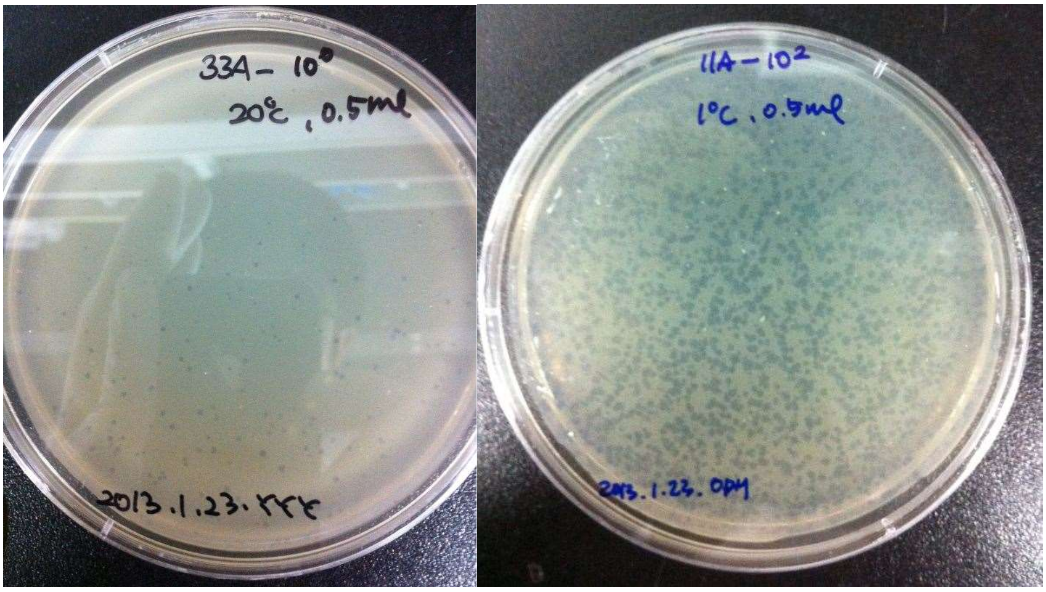

(a) (b)

Figure S1. Plaque images of ACA1 (a) and ACA2 (b) on marine agar plates

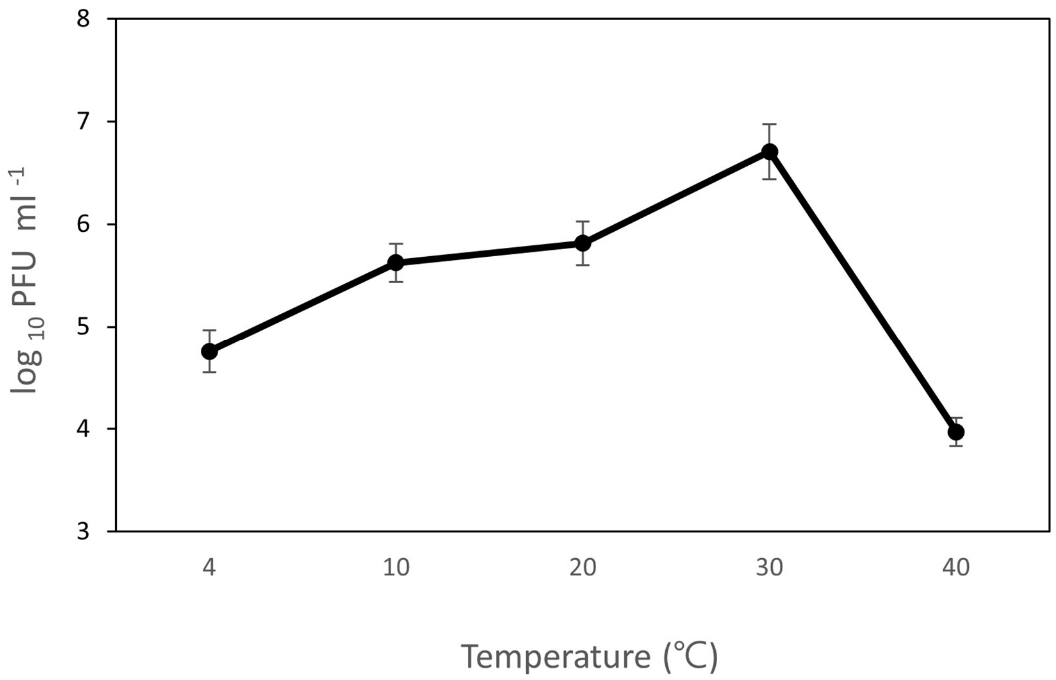

Figure S2. Phage production at different temperatures of phage ACA1. PFU; plaque forming unit

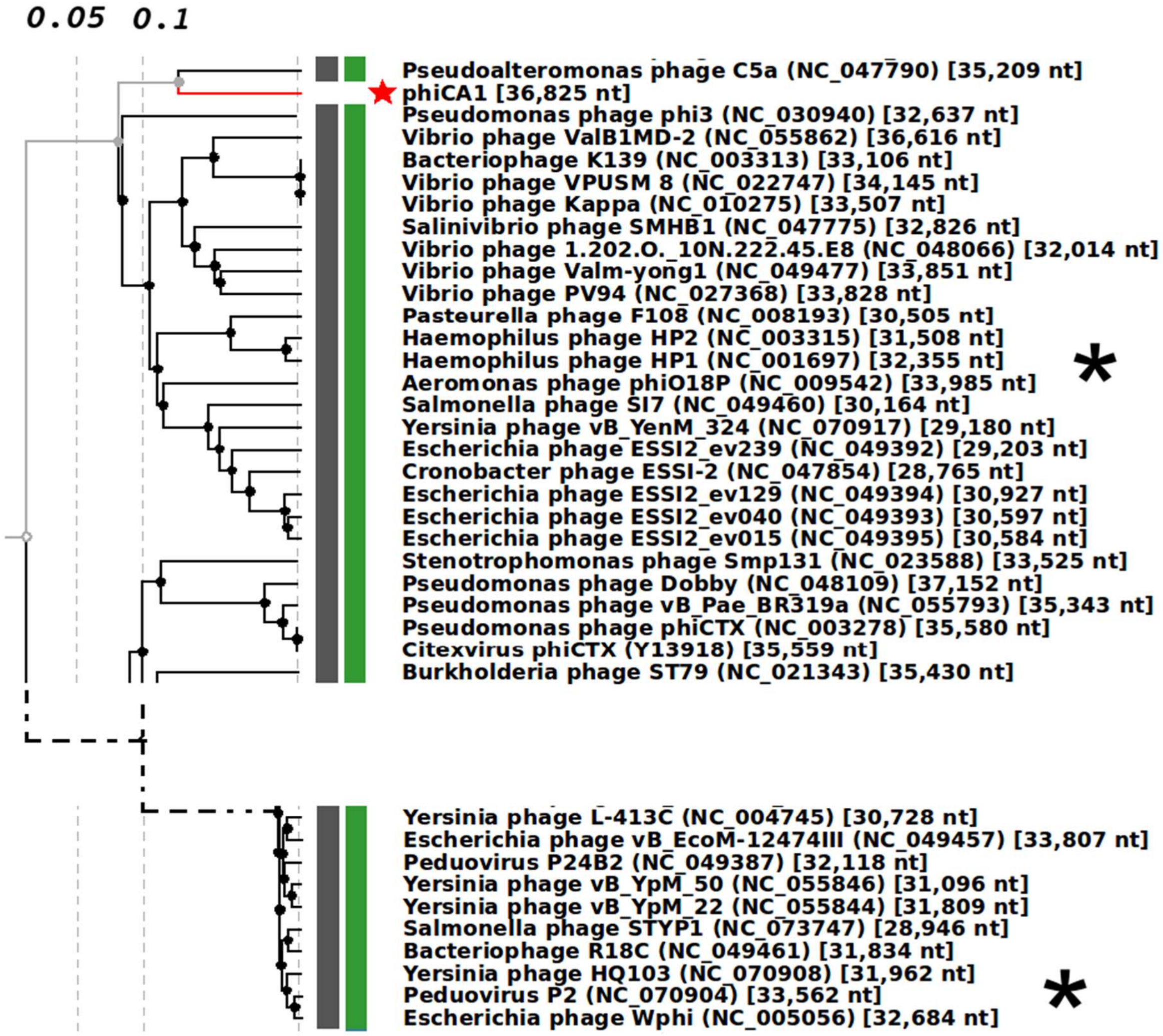

Figure S3. Segments from full proteome ViPtree with full reference sequence set showing placement of phage ACA1 near *Pseudoalteromonas* phage C5a, and within the HP1 subgroup within the larger *Peduoviridae* family. Prototypical phages HP1 and P2 representing the two large subgroups within *Peduoviridae* are marked with an asterisk.

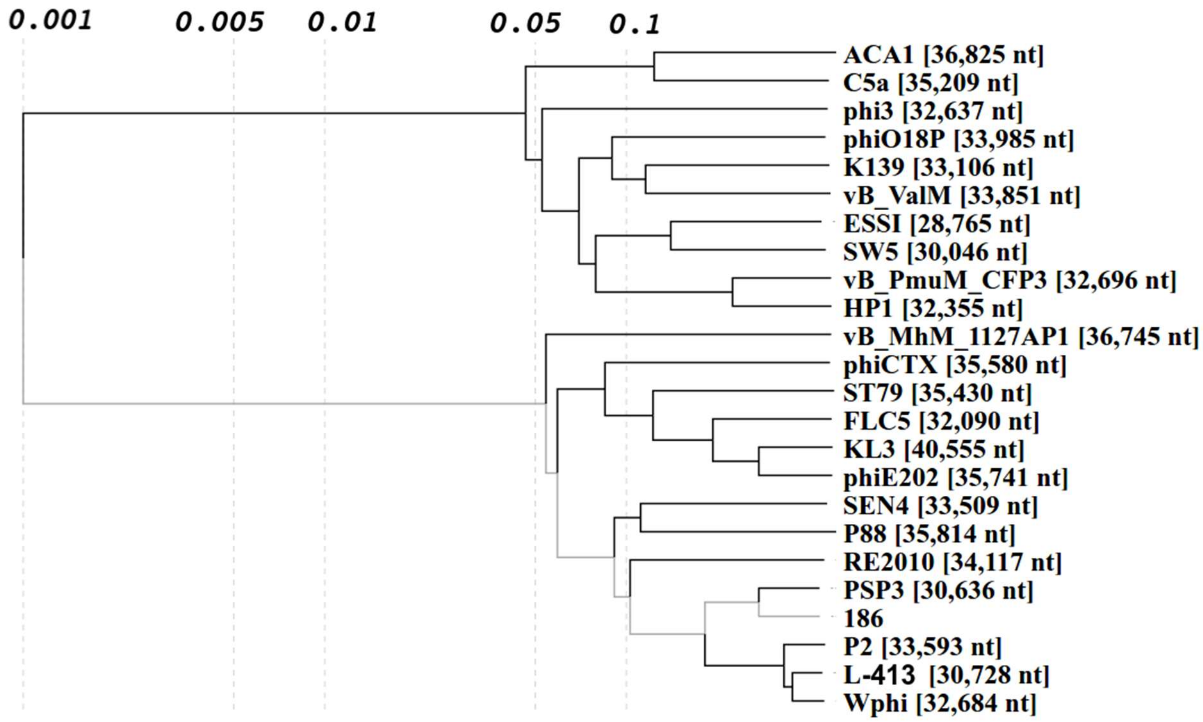

Figure S4. Whole proteomic ViPtree confined to just the exemplars used for single gene tree analysis in this paper. Other than the groups (P2, L-143, Wphi) and (PSP3, phage 186), all of these sequences represent different genera within *Peduoviridae*. Note that the split between the P2 and HP1 subgroups appears much deeper when done in this way, for reasons unknown.

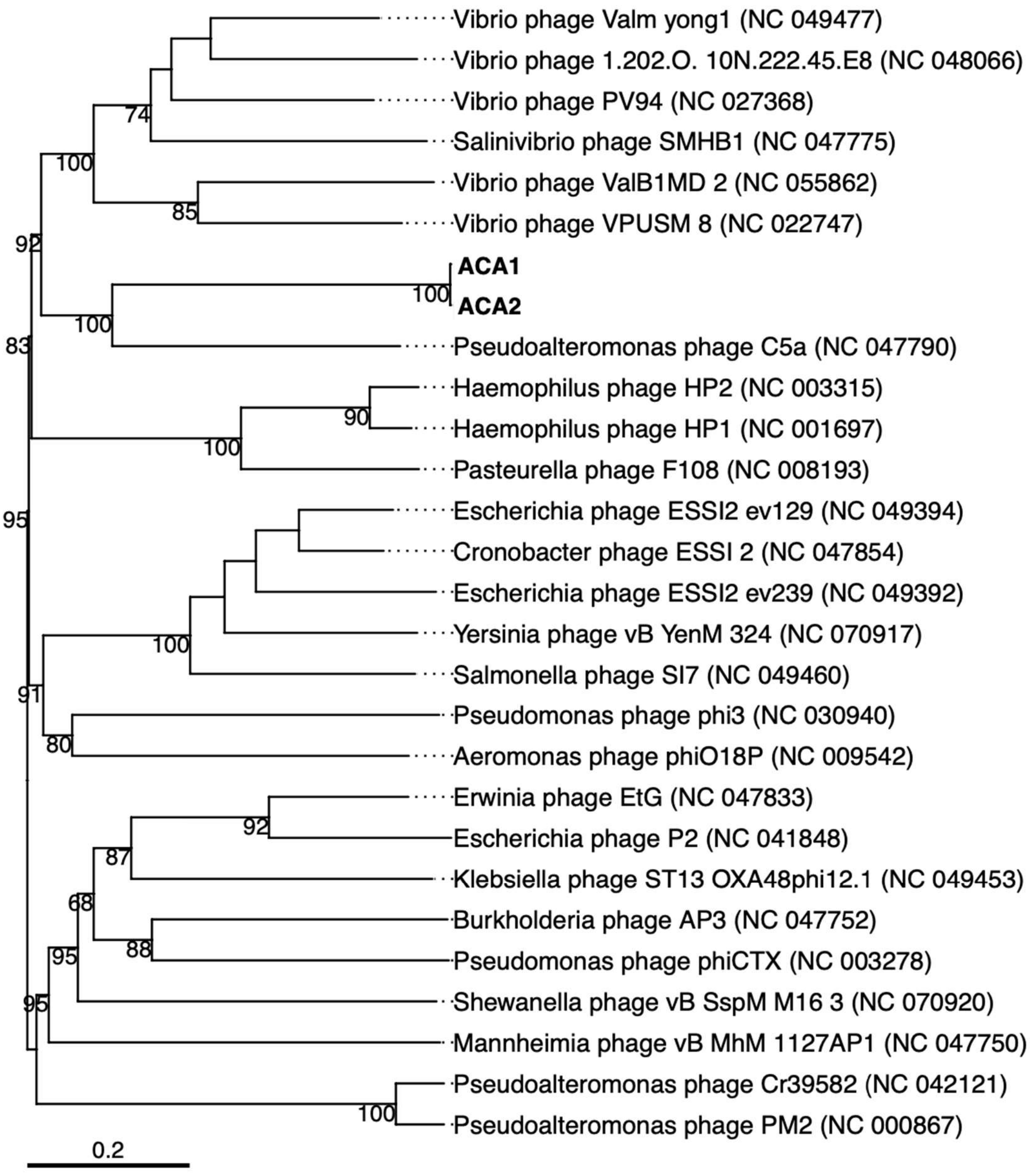

Figure S5. A whole genome-based VICTOR tree. The numbers above branches are GBDP pseudo-bootstrap support values from 100 replications.

Table S2. Mass spectroscopy of ACA1 virions.

Protein	Function	P2 homolog	MW (Kdal)	%coverage	SC[a]	SC/MW
gp25	portal	gpQ	37.1[b]	27	19[c]	0.51
gp27N	protease	gpO	16.1[b]	56	33	2.05
gp27C	scaffold	gpO	16[b]		n.d.[d]	
gp28	MCP	gpN	35.8	56	125	3.49
gp30	connector	gpL	15.9	18	4	0.25
gp31	connector	gpR	17.1	15	2	0.12
gp32	connector	gpS	17		n.d.	
gp33	sheath	FI	39	39	73	1.87
gp34	tail tube	FII	16.2	24	4	0.25
gp35	?		23.2		n.d.	
gp37	tape measure	gpT	122.2	22	24	0.2
gp38	baseplate	gpW	12.1	12	1	0.08
gp39	baseplate	gpJ	42.2	7	2	0.05
gp40	baseplate	gpI	30.1	17	7	0.23
gp41	tail fiber	gpJ	21.6	43	38	1.76
gp42	antireceptor		44.6	70	219[e]	4.91
gp46	HP1 orf33		22.6	6	2	0.09
gp47	baseplate?	gpU?	17.5		n.d.	
gp48	cell punct. device	gpD	26	15	5	0.19
gp49	cell punct. device	gpV	34.5	11	2	0.06

[a]Spectrum Counts. A spectrum is counted if assigned with 95% confidence.

[b]Molecular weight adjusted based on predicted maturation cleavage by comparison to P2 homolog.

[c]A semitryptic peptide was detected corresponding to the predicted maturation cleavage.

[d]Not detected. All other proteins were not expected to be detected, and were not detected. A few proteins are explicitly listed as not detected because there was some plausible hypothesis that they might be virion components.

[e]There were six spectra detected in the variable region; and they were all of the proACA1-A specificity.

Table S3. Phage genus and host of P2-like genomes used in the timetree analysis.

Phage	Genus	Host
**P2 subgroup**		
P2	*Peduovirus*	*E. coli*
phiE202	*Tigrvirus*	*Burkholderia*
KL3	*Kayeltresvirus*	*Burkholderia*
ST79	*Nampongvirus*	*Burkholderia*
FLC5	*Kisquattuordecimvirus*	*Burkholderia*
phiCTX	*Citexvirus*	*Pseudomonas*
Wphi	*Peduovirus*	*E. coli*
L-413C	*Peduovirus*	*E. coli*
186	*Eganvirus*	*E. coli*
PSP3	*Eganvirus*	*E. coli*
RE2010	*Felsduovirus*	*Salmonella*
P88	*Xuanwuvirus*	*E. coli*
SEN4	*Senquatrovirus*	*Salmonella*
vB_MhM_1127AP1	*Baylorvirus*	*Mannheimia*
**HP1 subgroup**		
HP1	*Hpunavirus*	*Haemophilus*
ACA1	not yet assigned	*Pseudoalteromonas*
C5a	*Catalunyavirus*	*Pseudoalteromonas*
Valm-yong1	*Yongunavirus*	*Vibrio*
phi3	*Phitrevirus*	*Pseudomonas*
vB_PmuM_CFP3	*Irtavirus*	*Pasteurella*
SW5	*Irrigatiovirus*	*Salmonella*
ESSI-2	*Seongnamvirus*	*Cronobacter*
phiO18P	*Bielevirus*	*Aeromonas*
K139	*Longwoodvirus*	*Vibrio*

Table S4. Similarity of selected *Campylobacter* phage PC10 structural proteins to P2 subgroup structural proteins.

Accession	Function	% identity[a] to P2 protein	% identity to a closer phage protein
**Tail proteins**			
QVW54053	sheath	22	
QVW54054	tail tube	21	
QVW54057	tape measure	17	
QVW54049	tail fiber (N-ter domain)	31	
UWJ04572	baseplate (gpI)	22	
**Head proteins**			
UWJ04524	portal	13	HK97 25%
UWJ04514	major capsid protein	11	HK97 32%

[a]Percent identity according to HHpred server.

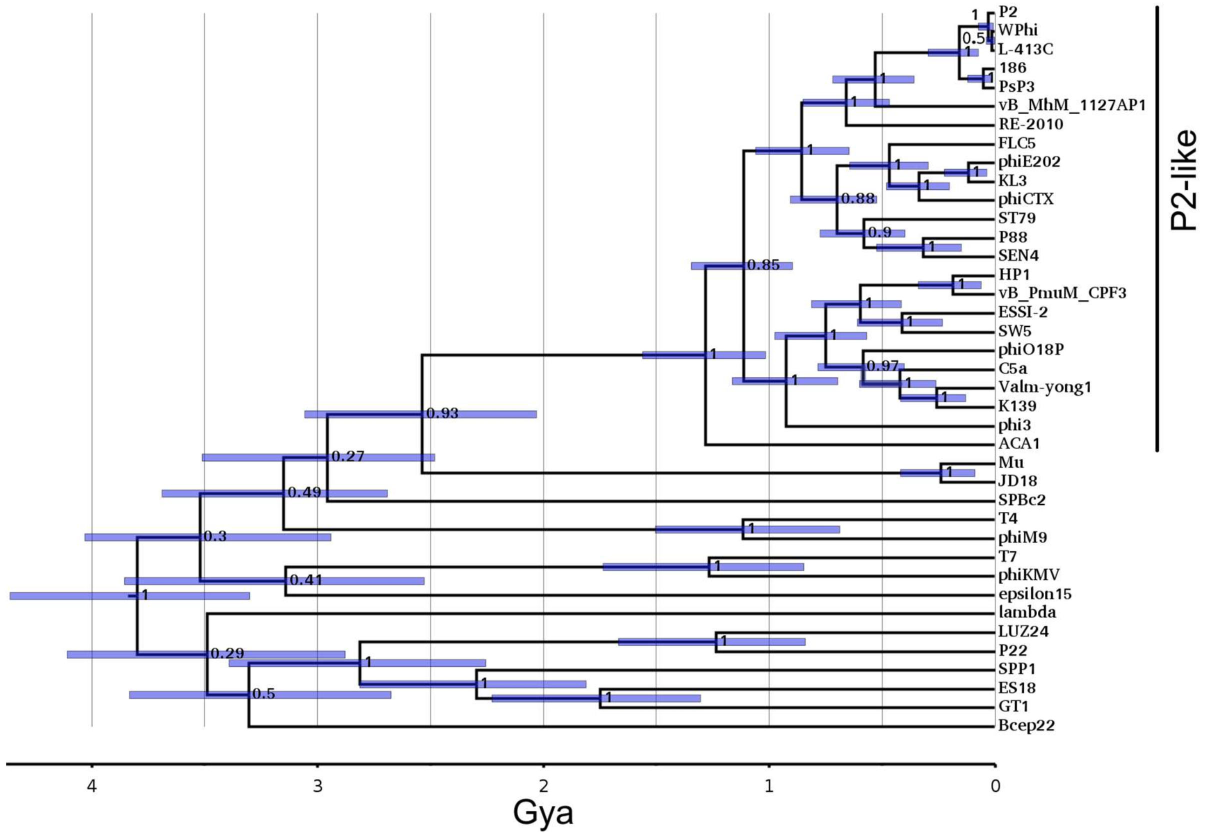

Figure S6. Timetree of P2-like large terminase proteins. The P2 phages used are summarized in Table S3. The time scale was formed by placing the root at 3.8 Gya. The tree is quite similar to the portal timetree.

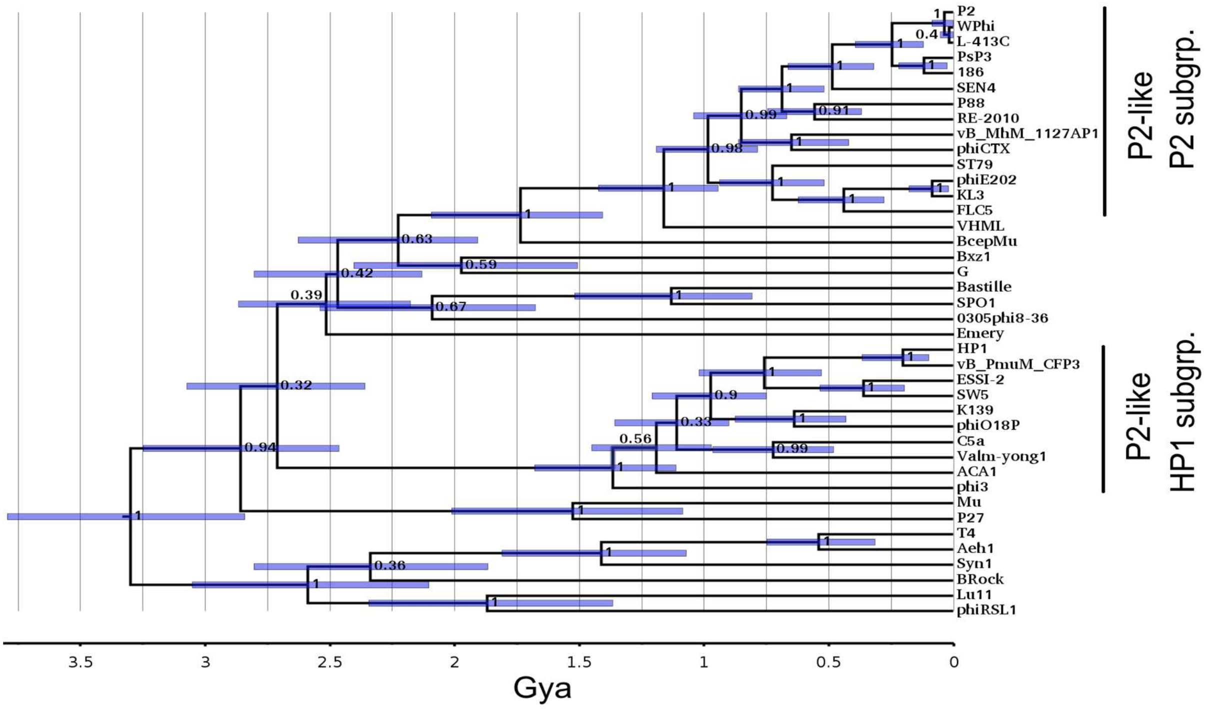

Figure S7. Timetree of P2-like gpJ baseplate proteins. The timescale was set by setting the radiation of the P2-like subgroup and the HP1-like subgroups to the same times as found for the sheath timetree. There is little evidence of recombination relative to the sheath tree within the P2-like phages. PC10, which had appeared high above the P2-like subgroup common ancestor does not have an identifiable homolog to this protein.

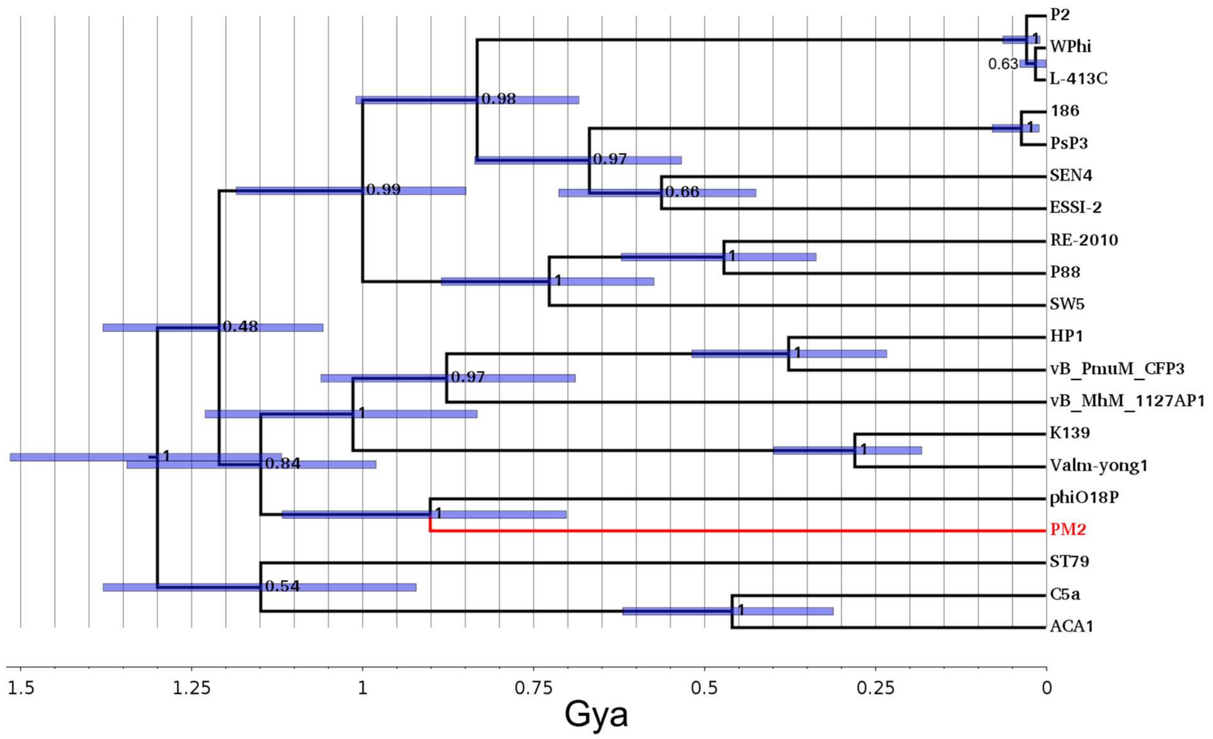

Figure S8. Timetree of P2-like repA proteins. This enzyme nicks the DNA to initiate replication. No homologs were found in non-P2-like tailed phages, although homologs in PM2 (in red) and related corticoviruses were found to be spawned from within the P2-like family. The P2 and HP1 subgroups are apparent, although there are some recombinations that exchanged this gene between the subgroups. Of the P2-like exemplars, FLC5, phiE202, KL3, phiCTX, and phi3 do not have homologs of this protein. This family is more distantly related to replication initiator proteins encoded in single stranded DNA phages and in some conjugative plasmids.

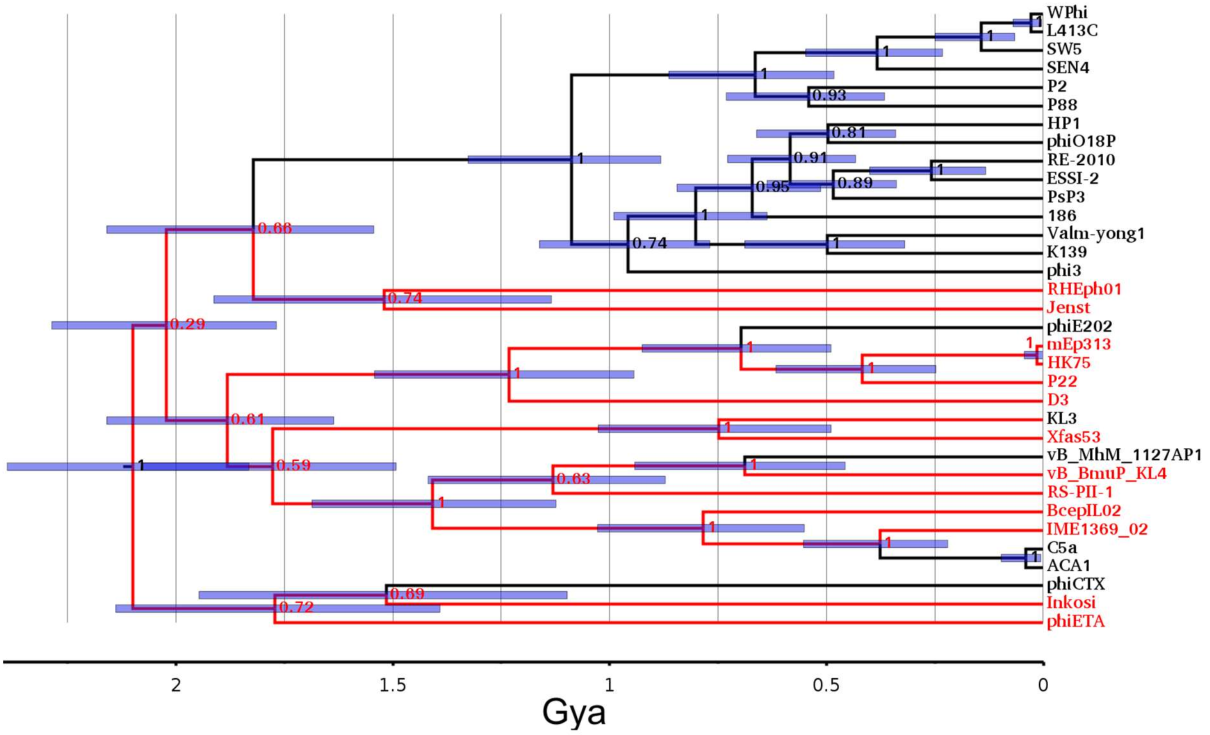

Figure S9. Timetree of integrases related to the ACA phages and P2-like phages. The integrase is among the most similar of the ACA phage proteins to *Pseudoalteromonas* C5a. P2-like phages are in black; the most closely related non-P2-like phages are in red. The time scale was set by placing the radiation of the main collection of P2-like phages at the same time as in the other trees. P2 and HP1 subgroups are still in evidence, but there is more extensive interchange between the subgroups than seen in the other trees. Some P2-like phages, including the ACA phages, have acquired a homolog from a different lineage than the one apparently supplying the common ancestor of the main P2-like group. In addition, of the P2-like exemplars listed in Table S3, FLC5 and ST79 did not have an identifiable homolog of this integrase.
